# Acetylsalicylic acid, aging and coronary artery disease are associated with *ABCA1* DNA methylation in men

**DOI:** 10.1186/1868-7083-6-14

**Published:** 2014-07-29

**Authors:** Simon-Pierre Guay, Cécilia Légaré, Andrée-Anne Houde, Patrick Mathieu, Yohan Bossé, Luigi Bouchard

**Affiliations:** 1 Department of Biochemistry, Faculté de médecine et des sciences de la santé, de l’Université de Sherbrooke, 3001 12e Avenue Nord, Sherbrooke, Québec J1H 5 N4, Canada; 2 ECOGENE-21 and Lipid Clinic, Chicoutimi Hospital, 305 rue St-Vallier, Saguenay, Québec G7H5H6, Canada; 3 Centre de Recherche Institut Universitaire de Cardiologie et de Pneumologie de Québec, Université Laval, 2725 chemin Ste-Foy, Québec, Québec G1V 4G5, Canada; 4 Department of Molecular Medicine, Université Laval, 2325 rue de l’Université, Québec, Québec G1V 0A6, Canada

**Keywords:** ATP-binding cassette transporter A1, Epigenetics, Aging, Cardiovascular disease

## Abstract

**Background:**

Previous studies have suggested that DNA methylation contributes to coronary artery disease (CAD) risk variability. DNA hypermethylation at the *ATP-binding cassette transporter A1* (*ABCA1*) gene, an important modulator of high-density lipoprotein cholesterol and reverse cholesterol transport, has been previously associated with plasma lipid levels, aging and CAD, but the association with CAD has yet to be replicated.

**Results:**

*ABCA1* DNA methylation levels were measured in leucocytes of 88 men using bis-pyrosequencing. We first showed that DNA methylation at the *ABCA1* gene promoter locus is associated with aging and CAD occurrence in men (*P* < 0.05). The latter association is stronger among older men with CAD (≥61 years old; n = 19), who showed at least 4.7% higher *ABCA1* DNA methylation levels as compared to younger men with CAD (<61 years old; n = 19) or men without CAD (n = 50; *P* < 0.001). Higher *ABCA1* DNA methylation levels in older men were also associated with higher total cholesterol (r = 0.34, *P* = 0.03), low-density lipoprotein cholesterol (r = 0.32, *P* = 0.04) and triglyceride levels (r = 0.26, *P* = 0.09). Furthermore, we showed that acetylsalicylic acid therapy is associated with 3.6% lower *ABCA1* DNA methylation levels (*P* = 0.006), independent of aging and CAD status of patients.

**Conclusions:**

This study provides new evidence that the *ABCA1* epigenetic profile is associated with CAD and aging, and highlights that epigenetic modifications might be a significant molecular mechanism involved in the pathophysiological processes associated with CAD. Acetylsalicylic acid treatment for CAD prevention might involve epigenetic mechanisms.

## Background

The ATP-binding cassette transporter A1 (ABCA1) catalyzes the transfer of lipids from various tissues and cells to apolipoprotein A1 containing lipoproteins [1]. This reaction is the rate-limiting step in the biogenesis of high-density lipoprotein particles and reverse cholesterol transport [1]. Mutations within the *ABCA1* gene in humans are responsible for Tangier disease (OMIM: 2054000) and familial hypoalphalipoproteinemia (OMIM: 604091) [2-4]. These two genetic disorders are characterized by markedly reduced plasma high-density lipoprotein cholesterol (HDL-C) levels, the accumulation of cholesterol esters in peripheral tissues and an increased risk of coronary artery disease (CAD) [2-6].

Previous candidate gene and genome-wide studies have suggested that DNA methylation contributes to CAD risk variability [7-13]. Indeed, we have recently shown that a higher DNA methylation level at the *ABCA1* gene promoter locus was associated with lower HDL-C levels and a previous history of CAD in familial hypercholesterolemia (FH) [7]. Moreover, our group and others have shown that higher *ABCA1* DNA methylation levels were associated with a lower *ABCA1* gene expression [14,15]. All these previous results suggest that perturbations of the *ABCA1* epigenetic profile might be a new molecular mechanism involved in CAD. However, these results have not yet been replicated.

DNA methylation is a non-traditional heritable factor occurring at cytosines located upstream of a guanine (CpG dinucleotides). It is involved in gene expression regulation [16]. This epigenetic modification is mitotically stable, and several environmental factors modulate its levels across the genome [16]. Interestingly, we recently observed that *ABCA1* DNA methylation level variability in newborns is associated with maternal glycemic and HDL-C status, suggesting that the *in utero* environment might modulate the *ABCA1* epigenetic profile [14]. Moreover, previous epigenetic studies performed by researchers from The Netherlands showed that aging and prenatal famine exposure are associated with DNA hypermethylation at the *ABCA1* gene promoter locus [17,18]. Overall, these results suggest that both the *in utero* and postnatal environments might modulate the *ABCA1* epigenetic profile and trigger a long-term susceptibility to cardiovascular diseases (CVDs) [14,17,18].

Environmental cardiovascular risk factors, such as smoking, a high-fat diet and physical activity, have been previously associated with DNA methylation variability in humans [19-22]. More interestingly, statins and acetylsalicylic acid (ASA), two drugs frequently prescribed to patients with a high cardiovascular risk profile, have been shown to be associated with the induction or attenuation of epigenetic marks *in vitro*[23,24]. However, no study has yet determined whether environmental cardiovascular risk factors or medication might be associated with the *ABCA1* DNA methylation profile in humans.

The aims of this study were thus to replicate the association between *ABCA1* DNA methylation and CAD in a non-FH population, as well as assess whether aging and environmental factors, especially tobacco smoking and medication, might be associated with *ABCA1* DNA methylation in a sample of 88 French-Canadian men.

## Results

**Figure 1:**
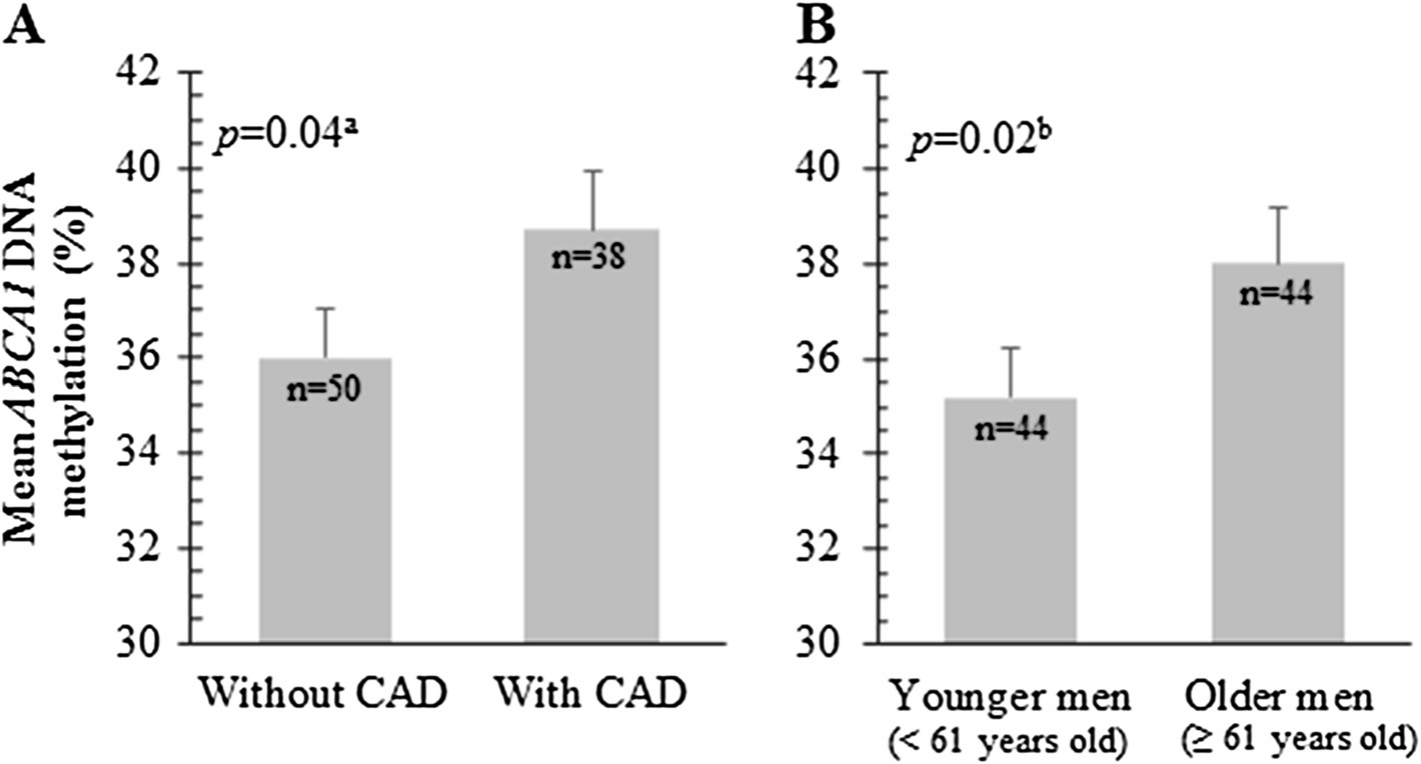
***ABCA1 *DNA methylation levels in leucocytes according to coronary artery disease occurrence and aging.** Men with a previous history of coronary artery disease (CAD) **(A)** and older men **(B)** showed higher mean *ATP-binding cassette transporter A1* (*ABCA1*) DNA methylation levels compared to men without history of CAD and younger men, respectively. ^a^*P*-values were obtained after consideration of patients’ current treatments and age. ^b^*P*-values were obtained after consideration of patients’ current treatments, CAD status and age.

Table 1 shows the characteristics of subjects according to their CAD status and median age (61 years old). We first assessed whether mean DNA methylation levels at 8 CpG dinucleotides located at the *ABCA1* gene promoter locus might be associated with CAD occurrence and aging in men (Figure 1). We observed that men with a previous history of CAD (n = 38) showed higher mean *ABCA1* DNA methylation levels than men without CAD (n = 50) (38.7 ± 1.2 versus 36.0 ± 1.0, *P* = 0.04; after consideration of age and current treatments) (Figure 1A). Moreover, older men (age ≥61 years old) had higher mean *ABCA1* DNA methylation levels than younger men (age <61 years old) (38.0 ± 1.2 versus 35.2 ± 1.0, *P* = 0.02; after consideration of CAD occurrence and current treatments) (Figure 1B).

**Table 1:** Characteristics of men according to their coronary artery disease status

**Characteristics**	**Younger men without CAD (n = 25)**	**Older men without CAD (n = 25)**	**Younger men with CAD (n = 19)**	**Older men with CAD (n = 19)**	***P*-value**	**Adjusted *P*-value**^**a**^
Age (years)	53.6 ± 1.4	72.6 ± 1.5	54.3 ± 1.9	73.4 ± 1.9	**<0.001**	-
BMI (kg/m^2^)	28.1 ± 1.1	27.0 ± 0.7	29.0 ± 1.2	26.3 ± 0.5	0.21	0.33
Waist circumference (cm)	100.4 ± 3.2	99.9 ± 1.7	102.2 ± 3.3	100.6 ± 1.7	0.94	0.81
Glucose (mmol/L)	5.12 ± 0.23	5.73 ± 0.20	6.00 ± 0.27	5.50 ± 0.20	0.06	0.06
Total cholesterol (mmol/L)	4.61 ± 0.20	3.78 ± 0.21	4.33 ± 0.30	3.91 ± 0.19	**0.03**	0.48
LDL-C (mmol/L)	2.58 ± 0.16	2.02 ± 0.17	2.67 ± 0.27	2.14 ± 0.16	**0.04**	0.63
HDL-C (mmol/L)	1.22 ± 0.08	1.16 ± 0.06	1.03 ± 0.05	1.12 ± 0.06	0.21	0.20
Fasting triglyceridemia (mmol/L)^b^	1.58 ± 0.37	1.22 ± 0.14	1.48 ± 0.12	1.33 ± 0.11	0.27	0.79
Smoking (ever/never)	15/10	14/11	14/5	9/9	0.50	-
Medication (%)						
ARB	4 (16)	5 (20)	5 (26.3)	4 (21.1)	0.89	-
ASA	6 (24)	9 (36)	11 (57.9)	16 (84.2)	**<0.001**	-
β-Blocker	8 (32)	7 (28)	9 (47.4)	11 (57.9)	0.16	-
Diuretic	8 (32)	10 (40)	3 (15.8)	5 (26.3)	0.36	-
Calcium channel blocker	2 (8)	6 (24)	9 (47.4)	7 (36.8)	**0.02**	-
Lipid-lowering drugs	8 (32)	17 (68)	12 (63.2)	18 (94.7)	**0.003**	-
ACE inhibitors	11 (44)	8 (32)	8 (42.1)	6 (31.6)	0.75	-
Nitroglycerine	1 (4)	4 (16)	5 (26.3)	9 (47.4)	**0.005**	-
Mean *ABCA1* DNA methylation (%)	36.1 ± 1.5	35.9 ± 1.5	33.9 ± 1.3	40.7 ± 2.0^c,d,e^	**0.04**	**<0.001**

Results are shown as mean ± SEM unless otherwise stated. Statistically significant results are shown in bold. ^a^*P*-values were obtained following an analysis of covariance including age and ASA as covariables. ^b^Geometric mean and *P*-value obtained after log10-transformation of the variable. ^c^*P* ≤ 0.05, compared to younger men without CAD following Bonferroni’s *post-hoc* comparison test; ^d^*P* < 0.05 compared to older men without CAD following Bonferroni’s *post-hoc* comparison test; ^e^*P* < 0.05 compared to younger men with CAD following Bonferroni’s *post-hoc* comparison test. *ABCA1*, ATP-binding cassette transporter A1; *ACE*, angiotensin-converting enzyme; *ARB*, aldosterone receptor blocker; *ASA*, acetylsalicylic acid; *BMI*, body mass index; *CAD*, coronary artery disease; *HDL-C*, high-density lipoprotein cholesterol; *LDL-C*, low-density lipoprotein cholesterol.

**Figure 2:**
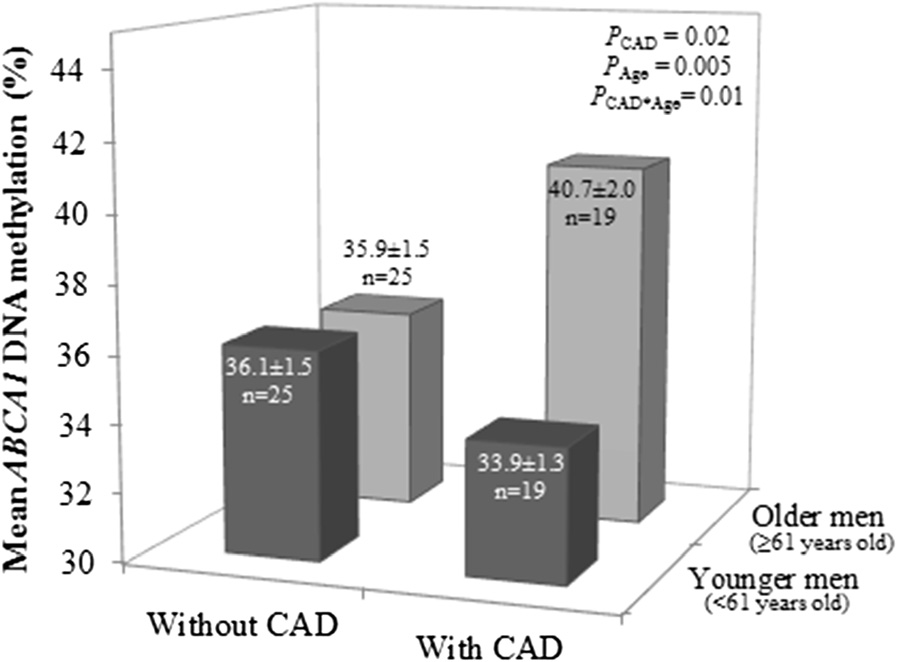
**Interaction between coronary artery disease and aging on *ABCA1 *DNA methylation levels in leucocytes.** Older men (>61 years old) with coronary artery disease (CAD) had the highest mean *ATP-binding cassette transporter A1* (*ABCA1*) DNA methylation levels. CAD occurrence and aging interact to increase *ABCA1* DNA methylation levels in leucocytes (*P* = 0.01). *P*-values were obtained after consideration of acetylsalicylic acid treatment.

Interestingly, we also observed a significant interaction between CAD status and age on mean *ABCA1* DNA methylation levels (*P* = 0.01; Figure 2). Older men with CAD (age ≥61 years old; n = 19) showed significantly higher DNA methylation levels at the *ABCA1* gene promoter locus compared to younger men without CAD (age <61 years old), older men without CAD (age ≥61 years old) and younger men with CAD (age <61 years old) (Table 1 and Figure 2), independently of current treatment. No significant mean *ABCA1* DNA methylation level difference was observed between younger men with or without CAD (*P* = 0.67). In older men (age ≥61 years old), we also observed that a higher *ABCA1* DNA methylation level was positively correlated with total cholesterol (r = 0.34; *P* = 0.03), low-density lipoprotein cholesterol (r = 0.32; *P* = 0.04), as well with fasting triglyceride levels (trend; r = 0.26; *P* = 0.09) after consideration of age, CAD status and medication. In younger men (age <61 years old), no significant association was observed between mean *ABCA1* DNA methylation levels and plasma lipid profile (data not shown).

**Figure 3:**
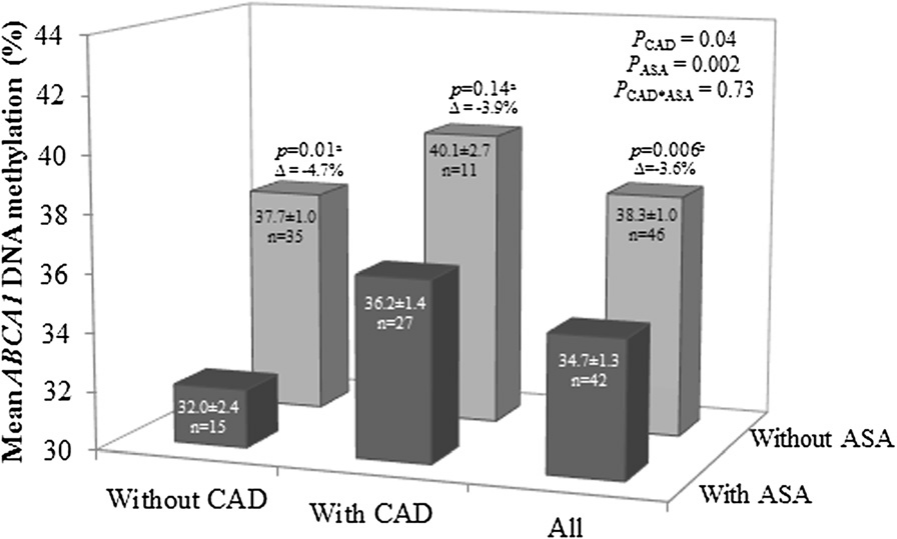
**Association between ABCA1 DNA methylation levels and acetylsalicylic acid therapy.** Subjects on acetylsalicylic acid (ASA) therapy had lower mean *ATP-binding cassette transporter A1* (*ABCA1*) DNA methylation levels (Δ = −3.6%) compared to subjects not on ASA therapy, independent of age and CAD. In subjects without CAD, we observed the same significant association between ASA and a lower mean *ABCA1* DNA methylation level (Δ = −4.7%; *P* = 0.01). Although not significant, we observed that in subjects with CAD, those under an ASA treatment exhibited a similar decrease in mean *ABCA1* DNA methylation levels (Δ = −3.9%; *P* = 0.14). ^a^*P*-values were obtained after consideration of age. ^b^*P*-value was obtained after consideration of age and CAD status.

Next, we further assessed whether smoking and medication were associated with the *ABCA1* epigenetic profile in men. No significant association was observed between *ABCA1* DNA methylation levels and tobacco smoking status (data not showed). However, we observed that subjects under ASA treatment (n = 42) had significantly lower mean DNA methylation levels at the *ABCA1* gene promoter locus compared to subjects not taking ASA (n = 46), even when the statistical model was adjusted for age and CAD (Figure 3). *ABCA1* DNA methylation levels measured at all eight CpGs were significantly associated with ASA treatment (all *P* < 0.008). No significant association was observed between *ABCA1* DNA methylation levels and the use of other medications (data not shown).

## Discussion

Previous work from our group and others has shown that *ABCA1* DNA methylation is associated with aging, dyslipidemia and CAD [7,18]. In the current study, we provide the first confirmatory evidence that a higher DNA methylation level at the *ABCA1* gene is independently associated with CAD, an association that might be specific to older men. We also provide additional validation of the independent associations with age and a proatherogenic lipid profile. As previously described, the relationship between dyslipidemia, CAD and higher DNA methylation levels at the *ABCA1* gene promoter locus (associated with a lower *ABCA1* gene expression) suggests that the ABCA1-driven reverse cholesterol transport is compromised when *ABCA1* DNA methylation increases [7,14,15]. This could lead to an increased susceptibility to CAD as seen in Tangier disease or familial hypoalphalipoproteinemia [2,4]. However, the higher *ABCA1* DNA methylation levels observed in older men with CAD could also be related to an increased severity of CAD or a longer disease history in these patients, but this relation will have to be confirmed. Nonetheless, the association between *ABCA1* DNA methylation and CAD that we first observed in FH subjects has now been replicated in men with common hypercholesterolemia.

Recently, results reported by Tobi and colleagues and our group also suggested that adverse fetal environmental conditions might have persistent epigenetic consequences on the *ABCA1* epigenetic profile and predispose newborns to develop obesity, diabetes and CVD [14,17]. However, it was additionally suggested that the postnatal environment might also modulate the DNA methylome [19-22]. The increased *ABCA1* DNA methylation that we observed with aging in men with CAD could thus illustrate the effects of an accumulation of exposures to pro-CAD environmental factors (such as a high-fat diet, physical inactivity or tobacco smoking) that induces *ABCA1* DNA methylation variability over time and predisposes to CVD. In the current study, we did not observe a significant association between the tobacco smoking status of men and *ABCA1* DNA methylation although smoking has already been associated with DNA methylation changes at other loci [19]. Unfortunately, it has not been possible to assess the association with other postnatal environmental exposures (diet or physical activity). Nevertheless, these results provide evidence in support of longitudinal and interventional studies.

Interestingly, we observed that subjects on ASA treatment had lower *ABCA1* DNA methylation levels compared to those not under this non-steroidal anti-inflammatory drug (NSAID) treatment. It has been previously suggested that ASA treatment is associated with a decrease of the concentration of atherogenic lipids, an increase of HDL-C levels, as well as the variability of gene expression levels of *ABCA1* and other key genes involved in reverse cholesterol transport in humans [25-28]. Previous epigenetic studies have shown that ASA and other NSAIDs might induce DNA methylation changes in humans [29-31]. Indeed, it has been suggested that ASA use might reduce the methylation rate associated with aging, especially at cancer-related genes [32]. More recently, Chang and colleagues suggested that ASA lowers the CAD risk by an epigenetic mechanism that protects against atherogenic electronegative low-density lipoprotein particles [23]. However, the mechanism by which ASA induces DNA methylation variability is still unknown, but likely involves its anti-inflammatory properties [30]. Indeed, inflammation has been known to promote *de novo* methylation, and ASA might therefore decrease DNA methylation levels at inflammatory-sensitive loci through its anti-inflammatory properties [30]. Based on these preliminary results and the current literature, it is tempting to speculate that ASA treatment lowers the CAD risk by reducing inflammation and *ABCA1* gene DNA methylation levels, leading in turn to the stimulation of the reverse cholesterol transport. Although this hypothesis will have to be confirmed in larger cohorts before strong conclusions can be drawn, our study brings new and promising research hypotheses to the fields of lipid research and pharmacoepigenetics.

One of the strengths of the study is the use of a robust and reproducible technology for DNA methylation quantification (that is, pyrosequencing of bisulfite-treated DNA). In addition, our sample size, although small for association studies, was fairly large compared with that of other epigenetic studies. Importantly, it allowed us to take into account possible confounding factors [11-13]. A limitation of this study relates to the impossibility to infer the causal relationship observed between *ABCA1* DNA methylation and CAD. Indeed, a patient’s methylome can either be modulated by CAD occurrence or be a molecular mechanism leading to CAD. Also, our study focused only on men in order to control for the possible confounding effects of sex hormones. It follows that our conclusions might not apply to women considering that the CAD risk profile might be associated with different epigenetically modulated loci in men and women. Finally, a candidate gene approach might seem less attractive compared to recent genome-wide methylation analyses [10,33]. However, we would like to point out that the *ABCA1* gene promoter locus analyzed in the current study is not assessed on the Infinium HumanMethylation27 and HumanMethylation450 BeadChips, the most widely used genome-wide methylation technologies. This clearly illustrates that candidate gene approaches are still essential, even in the current microarray era.

## Conclusions

This study confirms that higher DNA methylation levels at the *ABCA1* gene promoter locus are associated with aging and CAD in men. It supports the recently uncovered epigenetics-related cardioprotective effects of ASA treatment. It also provides the results of one of the few - though greatly needed - replication studies in epigenetic epidemiology. Overall, these findings could help our understanding of the molecular mechanisms involved in the pathophysiological processes leading to CAD in men and, in time, the development of new therapeutic strategies.

## Methods

### Sample and clinical data

This study included 88 men with (n = 38) and without CAD (n = 50) recruited from patients who underwent heart surgery at the Institut universitaire de cardiologie et de pneumologie de Québec (IUCPQ). All patients with and without CAD were caucasians (of French Canadian origin). Men with CAD were selected from patients undergoing coronary artery bypass grafting with a pre-operative angiogram showing the presence of at least one lesion of 50%. The average number of grafts per patient was 2.95 ± 1.55 (range 1 to 6). Only two patients had been diagnosed with acute coronary syndrome. Patients with a normal angiogram were selected as controls among individuals undergoing isolated valve surgery. All subjects were normoglycemic, and it was unlikely that they were FH based on their clinical history and the low prevalence of this inherited condition in the Quebec City area where they were recruited (prevalence of 1 FH subject on 208 inhabitants [34]. The subjects’ current treatments/medications (aldosterone receptor blocker, ASA, β-blocker, diuretic, calcium channel blocker, lipid-lowering drugs, angiotensin-converting-enzyme inhibitor and nitroglycerine), as well as their smoking status (current, former or never) were recorded. Patients signed an informed consent to include biological material and corresponding clinical data in our local biobank of the Centre de Recherche de l’IUCPQ (CRIUCPQ). This project received the approval of the IUCPQ ethics committee.

Fasting blood samples were obtained preoperatively. Plasma cholesterol, triglyceride and glucose concentrations were enzymatically measured using standard procedures.

### Nucleic acid extraction and DNA methylation level measurement

DNA was purified from buffy coat samples using the Qiagen QIAamp Blood Midi kit (Qiagen, CA, USA). *ABCA1* gene promoter DNA methylation levels were measured using pyrosequencing of sodium bisulfite-treated DNA, as previously described [7]. Briefly, this gold standard technology for DNA methylation analysis is a simple, accurate quantitative sequencing assay that combines sodium bisulfite DNA conversion, PCR amplification and sequencing by synthesis (PyroMark Q24, Qiagen). PCR and sequencing primers were selected using the PyroMark Assay Design v2.0.1.15 (Qiagen), as previously described [7] (Additional file 1: Figure S1). DNA methylation levels were measured at eight CpG dinucleotides upstream from the first exon of the *ABCA1* gene (*ABCA1*-CpG1 to -CpG8; Additional file 1: Figure S1). Considering that DNA methylation levels were found to be well correlated among these eight CpGs (r > 0.70; *P* < 0.001), a mean of *ABCA1* DNA methylation levels of these eight CpG dinucleotides was thus computed for each subject and subsequently used in the statistical analyses.

### Statistical analyses

The normal distribution of all variables was assessed using the Kolmogorov-Smirnov test. Only fasting triglyceride levels were not normally distributed and therefore log-_10_ transformed. Men were stratified into two groups according to the median age: younger men (age <61 years old) and older men (age ≥61 years old). Categorical variables were compared using a Pearson *χ*^2^-statistic, whereas mean differences between groups for continuous variables were compared with an analysis of covariance including the following potential confounding factors: age, medication and CAD status, and followed by Bonferroni’s *post-hoc* comparison test. Partial Pearson’s correlation was used to assess the association between *ABCA1* DNA methylation levels and plasma lipid concentrations after consideration of the same confounding factors. Results were considered statistically significant when *P*-values were <0.05 (two sided). All statistical analyses were performed with the IBM SPSS Statistic 20 software (release 20.0.0, SPSS, International Business Machines (IBM) Corp., NY, USA).

## Abbreviations

ABCA1: ATP-binding cassette transporter A1; ASA: acetylsalicylic acid; CAD: coronary artery disease; CRIUCPQ: Centre de Recherche de l’IUCPQ; CVD: cardiovascular disease; FH: familial hypercholesterolemia; HDL-C: high-density lipoprotein cholesterol; IUCPQ: Institut universitaire de cardiologie et de pneumologie de Québec; LDL-C: low-density lipoprotein cholesterol; NSAID: non-steroidal anti-inflammatory drug; PCR: polymerase chain reaction.

## Competing interests

The authors declare that they have no competing interests.

## Authors’ contributions

S-PG conceived the study design, participated in the data collection, performed the data analysis/interpretation and wrote the manuscript. CL performed data collection and revised the manuscript. A-AH contributed to data analysis/interpretation and revised the manuscript. YB and PM participated in the data collection, analysis and interpretation and revised the manuscript. LB conceived the study design, participated in the data analysis/interpretation process and revised the manuscript. All authors read and approved the final manuscript.

## Supplementary Material

Additional file 1: Figure S1 *ABCA1* gene promoter locus. The primary DNA sequence was numbered relative to the first *ABCA1* codon in exon 2 (Ensembl release 61 [February 2011]). The first exon of *ABCA1* is in red and bold type. The epigenotyped region is shown in green. Arrows indicate both PCR primer sequences. The analysed CpG dinucleotides have been numbered relative to the 5’ of the amplicon. Click here for file
