# Supplementary figures and images for: Acetylsalicylic acid, aging and coronary artery disease are associated with ABCA1 DNA methylation in men
Source: Clin Epigenetics. 2014 Jul 29;6(1):14. doi: 10.1186/1868-7083-6-14 (PMC4120725; doi:10.1186/1868-7083-6-14)

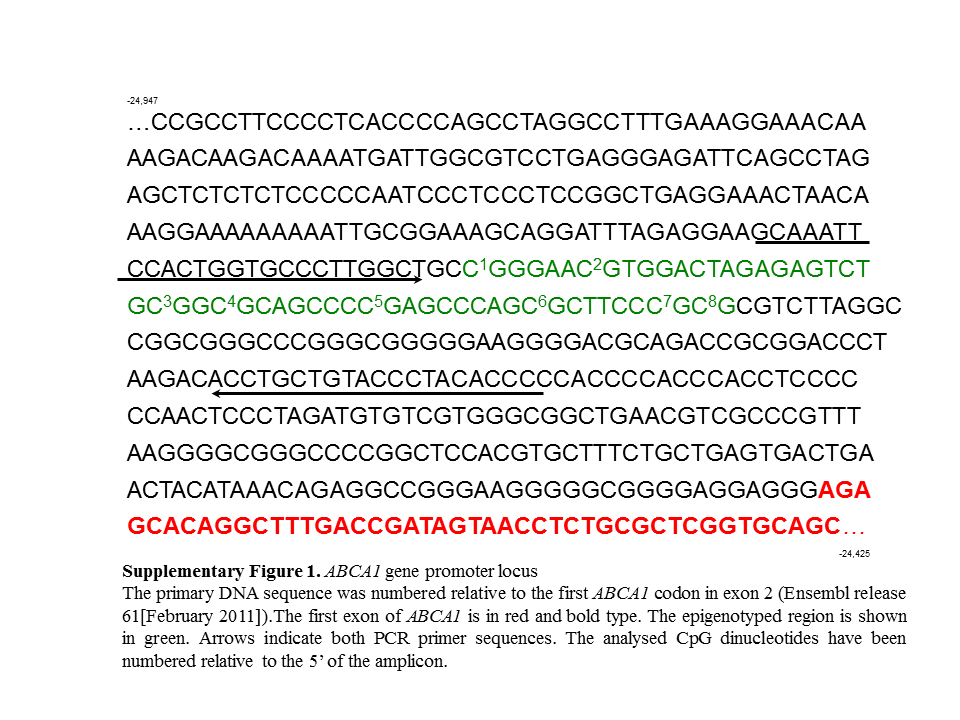

Supplement: Additional file 1: Figure S1 — ABCA1 gene promoter locus. The primary DNA sequence was numbered relative to the first ABCA1 codon in exon 2 (Ensembl release 61 [February 2011]). The first exon of ABCA1 is in red and bold type. The epigenotyped region is shown in green. Arrows indicate both PCR primer sequences. The analysed CpG dinucleotides have been numbered relative to the 5’ of the amplicon. [file 1868-7083-6-14-S1.tiff]
